# Predicting neoadjuvant chemotherapy benefit using deep learning from stromal histology in breast cancer

**DOI:** 10.1038/s41523-022-00491-1

**Published:** 2022-11-22

**Authors:** Fengling Li, Yongquan Yang, Yani Wei, Yuanyuan Zhao, Jing Fu, Xiuli Xiao, Zhongxi Zheng, Hong Bu

**Affiliations:** 1 Department of Pathology, West China Hospital, Sichuan University, Chengdu, China; 2 Institute of Clinical Pathology, West China Hospital, Sichuan University, Chengdu, China; 3 Department of Pathology, Shanxi Province Cancer Hospital/Shanxi Hospital Affiliated to Cancer Hospital, Chinese Academy of Medical Sciences/Cancer Hospital Affiliated to Shanxi Medical University, Taiyuan, China; 4 Department of Pathology, Sichuan Provincial People’s Hospital, Chengdu, China; 5 Department of Pathology, The Affiliated Hospital of Southwest Medical University, Luzhou, China

**Keywords:** Breast cancer, Risk factors, Predictive markers, Translational research

## Abstract

Neoadjuvant chemotherapy (NAC) is a standard treatment option for locally advanced breast cancer. However, not all patients benefit from NAC; some even obtain worse outcomes after therapy. Hence, predictors of treatment benefit are crucial for guiding clinical decision-making. Here, we investigated the predictive potential of breast cancer stromal histology via a deep learning (DL)-based approach and proposed the tumor-associated stroma score (TS-score) for predicting pathological complete response (pCR) to NAC with a multicenter dataset. The TS-score was demonstrated to be an independent predictor of pCR, and it not only outperformed the baseline variables and stromal tumor-infiltrating lymphocytes (sTILs) but also significantly improved the prediction performance of the baseline variable-based model. Furthermore, we discovered that unlike lymphocytes, collagen and fibroblasts in the stroma were likely associated with a poor response to NAC. The TS-score has the potential to better stratify breast cancer patients in NAC settings.

## Introduction

Neoadjuvant chemotherapy (NAC) is a standard treatment option for patients with locally advanced breast cancer and some large operable tumors^1,2^. In clinical trials, NAC has been shown to reduce the tumor burden and promote breast-conserving surgery, with patients who achieved a pathological complete response (pCR) having a better prognosis^3^. However, the pathological response rate varies among patients who receive this treatment modality and is primarily determined by their molecular subtype^4,5^. The heterogeneity of breast cancer in terms of the response to NAC has sparked renewed interest in predictive biomarkers, since these biomarkers facilitate clinical decision-making at the early stage.

Histological images contain a wealth of tumor phenotypic information and reflect the underlying molecular processes and disease progression, which can provide intrinsic information on diseases for the clinic. Subjective evaluation of pathological slides by well-trained pathologists is the gold standard for disease diagnosis and classification. However, pathological diagnosis mainly relies on visible morphological features, while the abundance of clinically relevant hidden information is currently not fully exploited. For instance, the Nottingham grading system provides prognostic and predictive information about breast cancer through pathologist assessment of histological features, including nuclear atypia, glandular differentiation, and mitotic count, but manual assessment can be subjective, is less reproducible and relies only on limited visible visual features. In recent studies, digital pathology and artificial intelligence (AI) techniques, which enable the extraction of hidden and quantitative information directly from histological images, have shown potential to provide clinically useful indicators^6,7^. In particular, the introduction of the convolutional neural network (CNN) has revolutionized the field of image analysis. Neural networks can distinguish objects by learning features from the training data and can effectively solve complex visual tasks^8^. Previous studies on digital pathology have used AI-based image analysis methods for tumor detection^9^, tumor grading^10,11^, immunohistochemistry (IHC) scoring^12^ and other medical classification tasks^13–15^, showing great potential in clinical application. More recently, deep learning (DL) methods based on medical images were used to develop novel biomarkers that were found to be predictive of the prognosis and chemotherapy response of patients^16–20^.

In a previous study, we proposed an image-derived biomarker for predicting pCR in breast cancer, which revealed hidden predictive information from the tumor epithelium^20^. Nevertheless, the tumor-associated stroma, also known as the tumor microenvironment (TME), has high potential for the discovery of novel biomarkers for predicting disease outcome. The tumor-associated stroma constitutes a suitable microenvironment for tumor growth, progression, and metastasis; the stromal phenotypic information presented on histology reflects the aggregate effect of underlying tumor biological alterations^21^. However, the high heterogeneity and complexity of the TME has hampered research progress on stroma-derived biomarkers from histological images. With the employment of AI techniques, several studies have found that stromal morphological features are predictive of prognosis in breast cancer^18^, prostate cancer^19^, and colorectal cancer^17^; in particular, Beck et al. proposed that the quantitative information extracted from the stroma was fairly predictive of prognosis in breast cancer^18^. Nevertheless, few studies have investigated the potential value of the stroma to predict the treatment response to chemotherapy. Although some stromal parameters from manual evaluation, such as tumor-infiltrating lymphocytes (TILs) and the tumor-stroma ratio, have shown some predictive ability for pCR^22–24^, abundant hidden information of the stromal morphology still remains to be exploited.

In this study, we aimed to fill this gap by exploring the potential value of tumor-associated stroma using AI techniques. We hypothesized that a stroma-derived biomarker could improve the prediction of pCR in breast cancer. We used DL-based methods to propose a stroma-derived biomarker from hematoxylin and eosin (HE)-stained histological images of breast cancer biopsies and evaluated the predictive power in four independent, multicenter datasets.

## Results

### Clinical characteristics

**Fig. 1:**
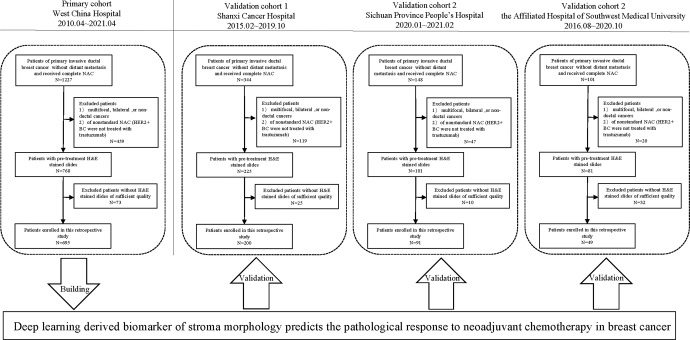
Patients recruitment and study design. 1035 patients out of 1820 with pretreatment H&E stained slides from four Chinese hospitals were included in this study for stroma-derived biomarker development and validation.

Figure 1 shows the workflow of patient recruitment. According to the inclusion and exclusion criteria, we enrolled a total of 1035 patients from four independent institutions: West China Hospital (WC cohort, 695 patients from 2010.04 to 2021.04), Shanxi Cancer Hospital (SX cohort, 200 patients from 2015.02 to 2019.10), Sichuan Province People’s Hospital (SC cohort, 91 patients from 2020.01 to 2021.02), and the Affiliated Hospital of Southwest Medical University (SW cohort, 49 patients from 2016.08 to 2020.10). The dataset from West China Hospital had the largest population of eligible patients (*N* = 695) and was used as the primary cohort (PC). The clinical characteristics of all patients are summarized in Table 1 (detailed information is available in Supplementary Table 4).

**Table 1:** Demographic and clinic-pathological characteristics.

	PC (*n* = 695)	V1 (*n* = 200)	V2 (*n* = 91)	V3 (*n* = 49)	*P*
Age at diagnosis					<0.001
<50	402 (57.8)	71 (35.5)	37 (40.7)	26 (53.1)	
≥50	293 (42.2)	129 (64.5)	54 (59.3)	23 (46.9)	
cT (%)					<0.001
T1–T2	334 (48.1)	164 (82.0)	57 (62.6)	30 (61.2)	
T3–T4	361 (51.9)	36 (18.0)	34 (37.4)	19 (38.8)	
cN (%)					<0.001
N0	58 (8.3)	35 (17.5)	27 (29.7)	16 (32.7)	
N1–N3	637 (91.7)	165 (82.5)	64 (70.3)	33 (67.3)	
HR status (%)					0.777
Negative	209 (30.1)	59 (41.8)	24 (26.4)	17 (53.1)	
Positive	486 (69.9)	141 (58.2)	67 (73.6)	32 (46.9)	
HER2 status (%)					<0.001
Negative	479 (68.9)	179 (89.5)	57 (62.6)	27 (55.1)	
Positive	216 (31.1)	21 (10.5)	34 (37.4)	22 (44.9)	
Subtype (%)					<0.001
HR+/HER2−	370 (53.2)	134 (67.0)	44 (48.4)	15 (30.6)	
HER2+	216 (31.1)	20 (10.0)	34 (37.4)	22 (44.9)	
TNBC	109 (15.7)	46 (23.0)	13 (14.2)	12 (24.5)	
Ki-67 index (%)					0.002
Low (<20%)	100 (14.4)	12 (6.0)	18 (19.8)	7 (14.3)	
High (≥20%)	595 (85.6)	188 (94.0)	68 (74.7)	42 (85.7)	
Unknown	–	–	5 (5.5)	–	
NG (%)					<0.001
1/2	486 (69.9)	162 (81.0)	79 (86.8)	42 (85.7)	
3	209 (30.1)	38 (19.0)	12 (13.2)	7 (14.3)	
sTILs (%)					0.195
Low	397 (57.1)	125 (62.5)	53 (58.2)	20 (40.8)	
Moderate	251 (36.1)	64 (32.0)	30 (33.0)	25 (51.0)	
High	47 (6.8)	11 (5.5)	8 (8.8)	4 (8.2))	
pCR (%)					<0.001
Yes	169 (24.3)	35 (17.5)	37 (40.7)	13 (26.5)	
No	526 (75.7)	165 (82.5)	54 (59.3)	36 (73.5)	

*HR* hormone receptor, *HER2* human epidermal growth factor receptor 2, *sTILs* stromal tumor-infiltrating lymphocytes, *NG* nuclear grade, *pCR* pathological complete response.

The pCR rates among the four cohorts were between 17.5 and 40.7% (Table 1). As shown in Supplementary Table 4, sTILs was significantly different between the pCR and non-pCR groups in all four cohorts (*P* < 0.05). In addition, pCR was associated with HR status and subtype in all cohorts except V3. Human epidermal growth factor receptor 2 (HER2) and nuclear grade were differentially distributed between the two groups in the PC and in one validation cohort (V2, V1). However, pCR was significantly correlated with Ki67 and cT only in the PC but not in the other three validation cohorts. We did not find a significant difference in age or cN between the pCR and non-pCR groups. Hence, subtype, nuclear grade, Ki67, and cT were baseline predictors of pCR, while sTILs was a strongly predictive factor manually evaluated from tumor-associated stroma.

### Automated detection of the stromal compartment

**Fig. 2:**
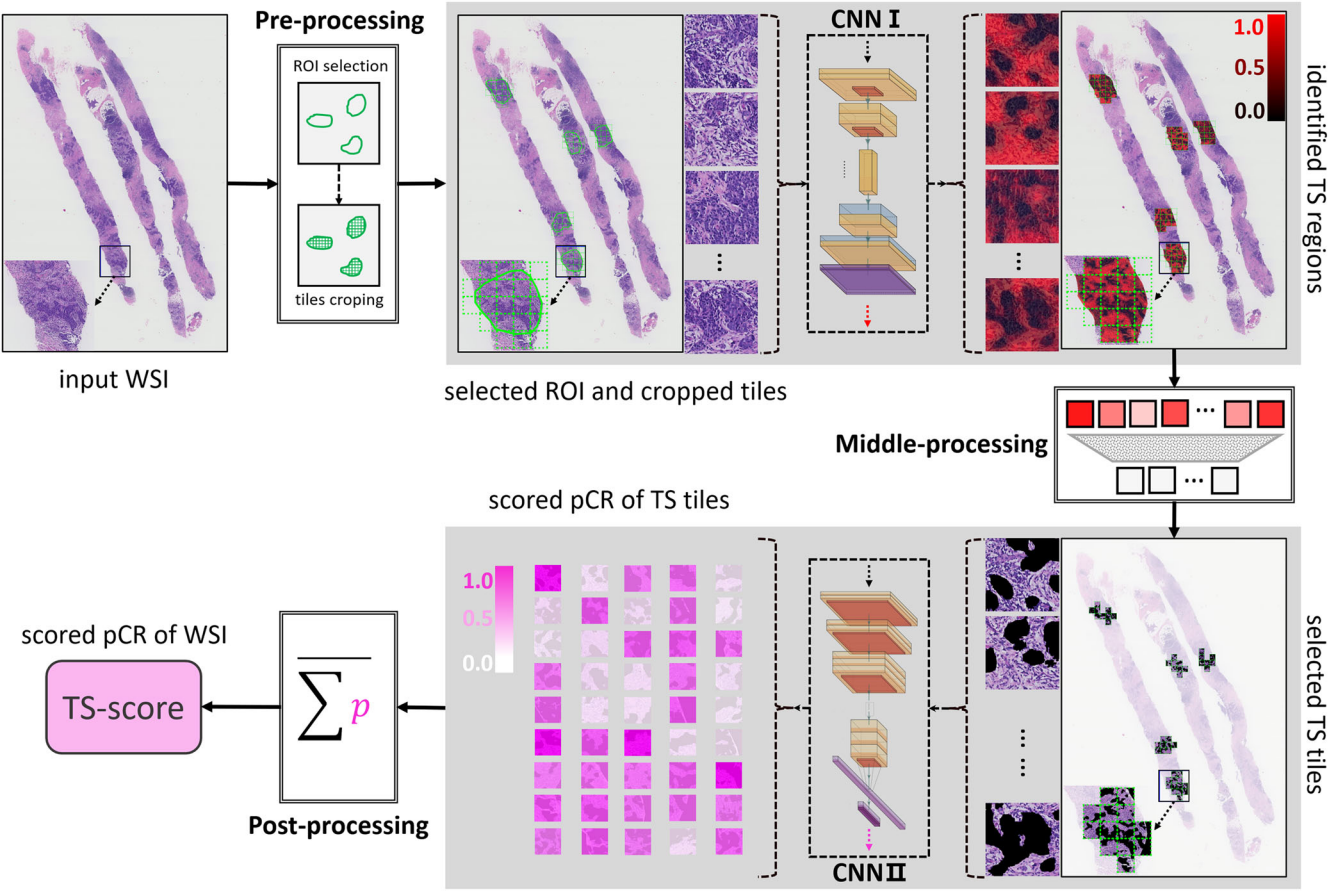
Image processing pipeline to develop a stromal-derived biomarker for predicting pCR. In the pre-processing step, the digitized HE-stained slides were manually annotated and the ROIs were cropped as tiles (256 × 256 pixels at 10 × magnification). With processed by the CNN I (also referring the E-S classifier), stromal pixels within the ROIs were detected and highlighted in red, and the color, red to black, indicates the probability of stroma from high to low. In the middle-processing step, a well-trained observer reviewed all tiles and removed the stromal tiles that did not exactly matched with the ground truth. Stroma tiles with identified by both the CNN I and the human observer were delivered to the CNN II and each tile was assigned with a score indicating the risk of achieving a pCR. In the post-processing step, all tile-level scores of each WSI were summed and the mean value was calculated and named TS-score, which was deemed as a DL-based biomarker derived from the stromal compartment and reflected the risk of pCR for breast cancer.

The epithelium-stroma classifier (E-S classifier) was applied to detect the stromal regions of all tiles cropped from the the region of interest (ROIs) of each whole-slide image (WSI). A total of 55,078 tiles were generated from 1035 WSIs. A heatmap of the stroma generated by CNN I is shown in Fig. 2 and Supplementary Fig. 2. As a result, the E-S classifier achieved the accuracy of 0.806 and 0.827 for stroma identification in the validation and testing sets (Supplementary Fig. 3 and Table 3), respectively. Furthermore, the E-S classifier showed high precision values of 0.896 and 0.870, which indicated that more than 85% of the area identified as stroma was exactly correct. After manual intervention, 44 stromal tiles per patient were enrolled on average. All remaining tiles were used in the following experiments of developing a stroma-derived predictor.

### TS-score construction and validation

**Fig. 3:**
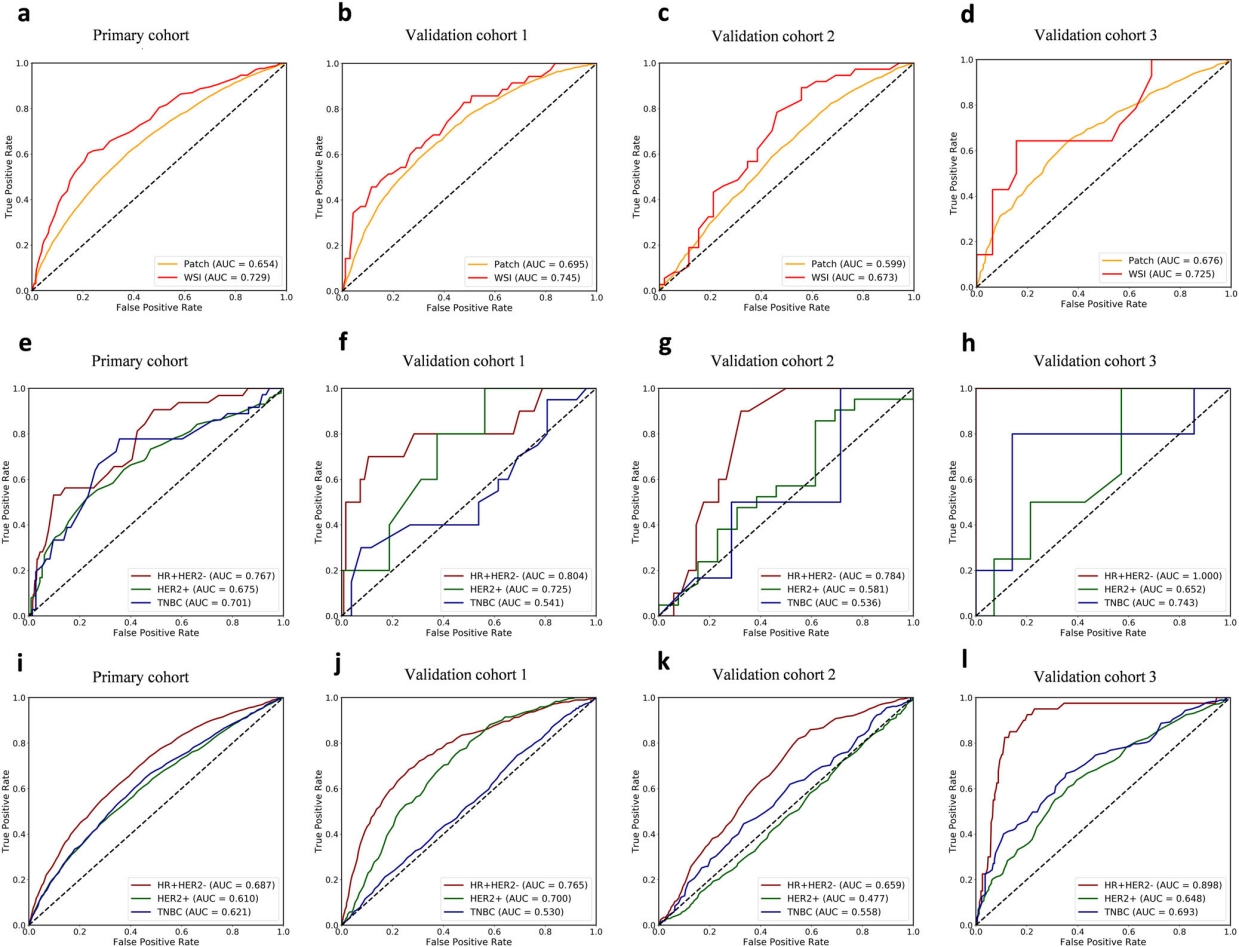
ROC curves of TS-score in the four hospitals. **a**–**d** Show the WSI-level and patch-level performance of TS-score in the total dataset among the four centers; **e**–**h** show the WSI-level performance of TS-score in different breast cancer subtypes, and **i**–**l** show the patch-level performance of TS-score in different breast cancer subtypes.

The construction pipeline of the TS-score is depicted in Fig. 2. A total of 44,734 stromal tiles with double certification from the E-S classifier and human observer were used. The Inception-V4 architecture was trained by learning from the stromal tiles with given labels of pCR or non-pCR in the PC, and 5-fold cross validation was used to determine the parameters of CNN II. After scoring all tiles, the TS-score of a given patient was obtained from calculating the mean value of the tile level, which reflected the predictive probability of obtaining pCR based on the tumor stromal compartment. The receiver operating characteristic (ROC) curves and areas under the curve (AUCs) of the raw TS-scores in the PC and three external validation cohorts are shown in Fig. 3. The TS-score achieved an AUC of 0.729 to predict pCR in the PC and AUCs of 0.745, 0.673, and 0725 in the V1, V2, and V3 datasets at the WSI level. Additionally, the TS-score showed stable performance in HR + HER2- breast cancer (AUC: PC 0.767, VC1 0.804, VC2 0.784, VC3 1.00), while the patch-level performance of the TS-score according to the three breast cancer subtypes is also shown in Fig. 3. Detailed results are available in Supplementary Tables 5 and 6.

### The TS-score is independent of clinical variables and improves the prediction of pCR

**Fig. 4:**
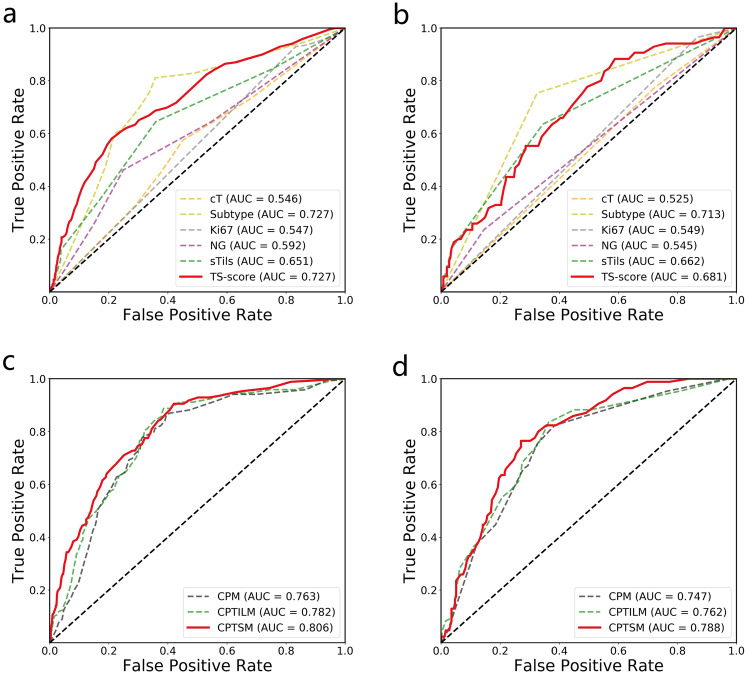
ROC curves of the marker-based models. The top row shows the performance of the single marker-based models for predicting pCR in the primary cohort (**a**) and the three external validations (**b**). The bottom row shows the performance of the baseline marker-based model (CPM), the baseline marker and sTILs-based model (CPTILM), and the baseline marker and TS-score-based model (CPTSM) for predicting pCR in three three external validations (**c**, **d**).

To evaluate the independent predictive power of the TS-score for pCR, we performed multivariate logistic regression analysis including factors that were potentially correlated with pCR (Table 2 and Supplementary Table 7) in the four datasets; due to the limited data size of the external validation cohorts, we combined the three validation cohorts to perform the following analysis. As shown in Table 2, the TS-score was significantly correlated with pCR in univariate analysis (*P* < 0.001), and it remained predictive when correcting for all other factors, including sTILs, subtype, T stage, Ki67, and nuclear grade (*P* < 0.001). Subtype was also significant (*P* < 0.001), but sTILs was not (*P* = 0.766), although sTILs was indeed a significant predictor in multivariate analysis without TS-score (*P* < 0.001). Similar results were observed in the external validation cohorts, as the TS-score was an independent predictor of pCR (*P* = 0.013) (Supplementary Table 7). Furthermore, using the logistic regression method, we developed factor-based prediction models of pCR to compare the predictive ability of the TS-score with other clinic-pathological (CP) factors (Fig. 4a, b). The TS score-based model yielded the best performance with an AUC of 0.727 in the PC, which was comparable to the subtype-based model (AUC = 0.727, *P* = 0.927) and even outperformed the sTIL-based model (AUC = 0.651, *P* < 0.001), and similar results were found in the validation cohorts. Detailed results are available in Supplementary Table 8.

**Table 2:** Univariate and multivariate analysis of TS-score correlating with pCR in primary cohort.

Factors	Univariate analysis		Multivariate analysis^a^		Multivariate analysis^b^	
	OR(95% CI)	*P*	OR(95% CI)	*P*	OR(95% CI)	*P*
TS-score	–	<0.001	–	–	–	<0.001
Subtypes	–	<0.001	–	<0.001	–	<0.001
HR+/HER2−	1	–	1	–	1	–
HER2+	9.28 (5.91–14.6)	<0.001	7.47 (4.66–12.0)	<0.001	7.73 (4.76–12.5)	<0.001
TNBC	5.21 (3.04–8.93)	<0.001	3.73 (2.10–6.62)	<0.001	3.33 (1.86–5.97)	<0.001
sTILs	–	<0.001	–	<0.001	–	0.766
Low	1	–	1	–	1	–
Moderate	7.58 (4.00–14.4)	<0.001	1.76 (1.16–2.69)	0.009	1.03 (0.64–1.66)	0.905
High	2.78 (1.47–5.25)	0.002	4.58 (2.20–9.54)	<0.001	1.36 (0.57–3.27)	0.490
cT	0.61 (0.43–0.86)	0.005	0.73 (0.49–1.10)	0.130	0.77 (0.51–1.16)	0.204
Ki67	2.63 (1.40–4.94)	0.003	1.40 (0.69–2.81)	0.348	1.19 (0.58–2.46)	0.636
NG	2.59 (1.80–3.70)	<0.001	1.37 (0.91–2.07)	0.137	1.21 (0.79–1.85)	0.372

Multivariate analysis ^a^refers to the multivariate analysis excluding TS-score; Multivariate analysis ^b^refers to the multivariate analysis including the TS-score.

*TS-score* tumor-stroma score, *HR* hormone receptor, *HER2* human epidermal growth factor receptor 2, *sTILs* stromal tumor-infiltrating lymphocytes, *NG* nuclear grade.

Univariate logistic regression analysis revealed that baseline CP factors, including subtype, nuclear grade, Ki67, and cT, were significantly predictive of pCR; sTILs and TS-score, stroma-derived factors, were predictive as well. Therefore, a CP-based model (CPM) combining subtype, nuclear grade, Ki67, and cT was constructed using the primary cohort; moreover, a model combining the factors above and the TS-score (CPTSM) was developed to evaluate the prediction incremental value of the TS-score. Additionally, a prediction model combining CP factors and sTILs (CPTILM) was built for comparison. As shown in Fig. 4c, the CPTSM yielded an AUC of 0.806 in the PC, while the CPM yielded an AUC of 0.763 and the CPTILM yielded an AUC of 0.782. Using the DeLong test, the CPTSM showed a significantly higher AUC than either the CPM (*P* < 0.001) or the CPTILM (*P* = 0.005) (Table 3). Similar results were also observed in the external validations; the CPTSM significantly outperformed the CPM (*P* = 0.027) and showed a higher AUC than the CPTILM, which was close to significance (*P* = 0.078) (Fig. 4d and Table 3). The results for VC1, VC2, and VC3 are available in Supplementary Fig. 6 and Table 9.

**Table 3:** AUCs of models for pCR prediction in the primary and validation cohorts.

	CPTSM (95% CI)	CPTILM (95% CI)	CPM (95% CI)
PC	0.806 (0.78–0.83)	0.782 (0.73–0.83)	0.763 (0.72–0.81)
* p* value^1^	<0.001	0.04	1
* p* value^2^	0.005	1	–
VCs	0.788 (0.783–0.793)	0.762 (0.756–0.768)	0.747 (0.742–0.752)
* p* value^1^	0.027	0.180	1
* p* value^2^	0.078	1	–

*p* value refers to Delong test for the differences of AUCs between different metrics in different cohorts; *p* value^1^ refers the comparisons with the CPM while *p* value^2^ refers the comparisons with CPTILM.

*AUC* area under receiver operating characteristic curve, *pCR* pathological complete response, *CPM* clinicopathology-based model, *CPTILM* clinicopathology and sTILs based model, *CPTSM* clinicopathology and TS-score based model.

### The TS-score reflects the stromal histological patterns that correlate with pCR

**Fig. 5:**
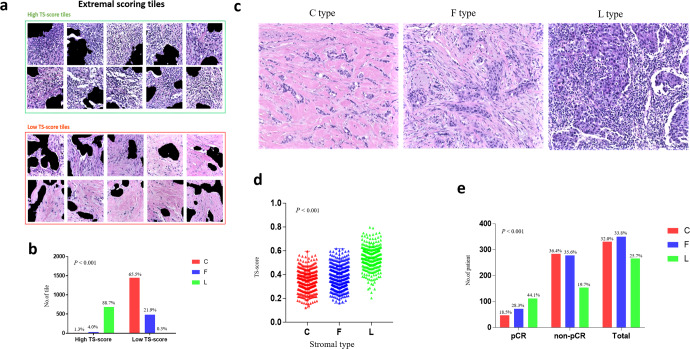
TS-score reflects the stromal histological patterns associated with the pCR. The underlying histological patterns of TS-score characterizing at the patch-level (**a**, **b**) and WSI-level (**c**–**e**). **a** Tiles with extremal TS-scores associated with pCR and non-pCR were extracted to be reviewed by a pathologist. Scale bars, 233 μm. **b** The distribution of tiles with different stromal type between extremal high TS-score and low TS-score group. **c** Examples of different stromal type: collagen-dominant stromal (C type), fibroblast-dominant stroma (F type), lymphocyte-dominant stroma (L type). Scale bars, 100 μm. **d** The distributions of TS-scores among the three stromal type evaluated at WSI-level. **e** The different percentage of the three stromal type between pCR and non-pCR group.

To obtain an overall understanding of the histological patterns that contribute to the exact prediction, the distributions of each tile score in the PC were visualized, and the tiles corresponding to extremal scores (top 10% and bottom 10%) were extracted for manual evaluation. These extremal patches (*n* = 2980) were classified into three stromal phenotypes, which were the collagen-dominant type (C type), fibroblast-dominant type (F type), and lymphocyte-dominant type (L type) [27]; tiles that did not belong to any of the three types were excluded from the analysis (Fig. 5c). High TS-score tiles were mainly L-type tiles, while C-type stromal tiles were few (684/771, 10/771). In contrast, low TS-score tiles mainly showed rich collagen or partly had a higher distribution of F-type stroma and an extremely low percentage of L-type stroma (1447/2209, 484/2209, 7/2209). A significant difference was shown among the distributions of stromal type between tiles of the highest 10% and lowest 10% TS-score (*P* < 0.001) (Fig. 5a, b), as high scores were mostly predicted based on lymphocyte-dominant regions and low scores were mostly predicted based on collagen-dominant stroma.

To further determine the relationship between stromal histological patterns and the treatment response to NAC, we also assessed the stromal type of 1035 HE-stained images among the four hospitals at the WSI level. In the pCR group, L-type stroma was dominant (44.1%), while patients with C-type and F-type stroma were more common in the non-pCR group (36.4 and 35.6%, *P* < 0.001) (Fig. 5e). Correspondingly, patients with L-type stroma showed the highest TS scores, followed by those with F-type stroma, and those with C-type stroma had the lowest TS-scores (*P* < 0.001) (Fig. 5d). Additionally, the pCR rates were 14.2, 20.6, and 42.1% in patients with C-type stroma, F-type stroma, and L-type stroma, respectively (Supplementary Table 10).

## Discussion

In this study, we proposed a new stroma-derived biomarker, the TS-score, and investigated its predictive ability for treatment response to NAC with a multicenter dataset. Experiments showed that the TS-score is predictive of pCR independent of subtype, tumor size, sTILs, nuclear grade, and Ki67, which can provide complementary information for predicting pCR, outperforming routine CP biomarkers. According to the histological patterns reflected by the TS-score, interestingly, we discovered that stroma with collagen and fibroblast dominance was likely associated with an inadequate response to NAC, which was contrary to the lymphocyte-dominant stroma. We also assessed the stromal type on WSIs and further identified this relationship between stromal histological patterns and pCR at the patient level. In summary, the TS-score, which is directly obtained from routine HE-stained images, can serve as a potential candidate to improve the prediction of pCR in breast cancer.

Our study investigated the DL-based prediction of pCR to NAC in breast cancer, a disease with the highest incidence in females and with wide variations in the treatment response to NAC^25^. Treatment planning for breast cancer is dependent on several factors, such as clinical stage and molecular subtype. However, due to their limited predictive ability, the field of breast oncology is currently awaiting features that can better distinguish chemotherapy sensitivity and chemotherapy resistance. In our study, the TS-score outperformed the baseline predictors in predicting pCR and performed as well as molecular subtype in the primary and external validation cohorts, despite using only a very small portion of each histological image. Remarkably, the TS-score even outperformed sTILs even though both are stromal histological predictors of pCR. Given that histological assessment of sTILs has been limited by considerable intra- and interobserver variability, the TS-score can effectively extract the predictive information from histological images via a highly reproducible and quantitative approach that compensates for the defect of sTILs. Additional investigations of the independence of the TS-score revealed that it can provide complementary information to the baseline factors for predicting pCR, and the comparisons of models demonstrated that the addition of the TS-score can improve the prediction performance with statistical significance, which is meaningful because improving the prediction performance can facilitate favorable patient care in NAC settings.

In breast cancer, the potential ability of tumor-associated stroma was investigated via manual pathological evaluation^22–24,26^, molecular biological assays^27,28^, and digital pathological techniques^18,29^, showing that the stroma could facilitate disease classification and outcome prediction. However, to our knowledge, the potential information in the stromal compartment has not been mined to predict pCR by DL techniques. Therefore, our study constitutes a precedent for objectively assessing hidden information from the stroma and proposing a stroma-derived biomarker to improve the prediction of pCR in breast cancer. Conventionally, pathological diagnosis is based largely on the histological appearance and molecular characteristics of epithelial cells, while stromal alterations are often subtle and difficult to characterize and quantify by manual evaluation. Moreover, the tumor stroma is highly heterogeneous and complex in breast cancer, which could be challenging for ROI selection and automated identification of the tumor stroma. Hence, manual annotation was required in our study to select representative regions, as in a previous study in prostate cancer^19^. Unlike automated detection for tumor epithelium^20^, stroma identification is a more difficult task; hence, we employed state-of-the-art algorithms and used a larger sample size to construct an improved model to detect the stromal pixels within ROIs. Unlike Kather and colleagues^17^, who quantified the various components of the stroma in colorectal cancer and combined them into a stroma score, the CNN II in our study learned directly from the stromal compartment, integrating the predictive information into a biomarker: the TS-score. Compared to their study, an end-to-end approach to extract information is likely simpler and more likely to discover the hidden interaction patterns between different components despite the weakened interpretability.

Another important aspect of the TS-score is its interpretability. As in all studies using DL-based methods, one question always arises: what exactly does the output score represent? Essentially, many DL-based models are complicated neural networks that may have tens to thousands of layers^30^, making it challenging to interpret their predictions in a way that humans can understand. This is crucial, however, as these will be widely used and supported only when the underlying decision process can be understood. In fact, we have tried to present more explanations for the predictions of our DL approach from the pathological perspective, similar to the study of Courtiol et al^14^. Primarily, by visually examining the predictions of our DL approach, we discovered that the predictions of our DL-based approach seems to be able to reflect some stroma-histological patterns which have been shown to be correlated with the prognosis of breast cancer in a previous study^31^. Subsequently, on the basis of this discovery, we further tried to interpret the predictions of our DL-based approach with existing pathological knowledge, which revealed that the output score (TS-score) of our DL-based approach shows diversity in different stromal types both on patch level and WSI level. Finally, referring to the revealed diversity of TS-score in various stromal types, we conducted statistical analyses and manual assessments based on stroma-histological types. The results have shown to be correlated with treatment response in breast cancer (Fig. 5), which proves that the predictions of our DL-based approach not only reflect some existing prior pathological knowledge^22,23^ but also can provide new insights, which may not be noticed before, for predicting treatment response to NAC from stroma histology. For instance, the explanations suggested that breast cancer with collagen and fibroblast-dominant stroma may have a high risk of failing to achieve pCR, but these histological patterns have not yet been widely acknowledged as characteristics of chemo-resistance behavior or taken into account in pathological evaluation paradigms. Therefore, even though the offered explanations for the predictions of our DL method from the pathological side in this study are far from being complete theoretical explanations, they have strengthened our confidence in DL techniques for medical decision-making.

In fact, AI technique-based image analysis has broad applications in modern medicine. In radiology, some DL-based inventions have already been approved by the FDA^32,33^. Compared to these imaging modalities, histological images contain more abundant information and provide the gold standard for diagnosis; combining AI techniques has promising prospects for clinical use. The clinical translation of digital pathology, however, is still in its infancy. To advance clinical applications, large amounts of training data and robust multicenter validation are needed, while many current studies are hampered by these limitations. In the present study, we addressed these limitations: four independent datasets of more than one thousand cases were used to establish and validate the CNN-based TS-score as a predictive biomarker in breast cancer. With this approach, we showed that the DL-based stromal score improved the prediction of pCR in breast cancer. Furthermore, by validating it in three external datasets, we confirmed the predictive potential of this approach. Therefore, we presented a novel candidate for NAC response prediction, which could be combined with existing predictors to better stratify patients and facilitate clinical decision-making.

The study had some limitations. Although 1035 breast cancer patients were recruited from four hospitals, the size of the validation dataset was limited, with two validation cohorts including fewer than 100 patients. Furthermore, we only included retrospective data, and this study needs to be validated prospectively.

Despite these limitations, our study is the first to show the potential ability of the breast cancer stromal compartment in pCR prediction via a DL-based approach. Furthermore, the findings of this study provide some insight into the different characteristics of the TME between pCR and non-pCR breast cancer patients. Future work will need to replicate and validate these findings in larger cohorts and prospective clinical trials. In addition, we will continue our studies on the spatial patterns between the tumor epithelium and stroma to further explore the potential of breast cancer histology.

## Methods

### Study design

Based on a multicenter study of 1035 breast cancer patients from four independent Chinese hospitals, a new biomarker, called TS-score, directly derived from the tumor stromal compartment, was proposed to predict the treatment response to NAC in patients with primary invasive breast cancer. Histopathological assessment of the resected breast specimens after surgery was used as the reference standard, and the TS-score was compared with baseline CP variables and manually evaluated TILs derived from the tumor stroma. The predictive incremental value of the TS-score for predicting pCR was also evaluated using the CP variable-based model as the reference baseline. In addition, we explored the potential histological patterns of the breast cancer stroma that the TS-score characterized. Our study was approved by the ethics committee of each participating hospital and abided by the Declaration of Helsinki before using tissue samples for scientific research purposes only. The requirement to obtain informed consent from the participants was waived by the ethics committee.

### Patients

The inclusion criteria were as follows: (1) patients with primary invasive ductal breast cancer; (2) patients without distant metastasis; (3) patients receiving four, six, or eight cycles of anthracycline and/or taxane-based NAC regimens, and patients with HER2+ diseases who underwent targeted HER2 therapy (NAC regimens are detailed in Supplementary Table 2); and (4) patients who had undergone surgical treatment after NAC. On the other hand, patients with HE-stained slides of poor quality, including tissue-processing artifacts such as bubbles, discoloration, and soiling caused by long storage time and low tissue volume, were excluded from our study. In total, 1035 eligible patients were enrolled, and a detailed recruitment flowchart is shown in Fig. 1 and the criteria were in Supplementary Table 1. The dataset with the largest population was assigned as PC for developing the image-derived predictor, and the other three cohorts were used as validation cohorts (V1–V3).

Our study approved by the ethical committee of West China Hospital, Sichuan University (No.764 in 2021), and abided with the Declaration of Helsinki before using tissue samples for scientifc researches purpose only. The other three hospitals, including Shanxi Cancer Hospital, Sichuan Province People’s Hospital, and the Affiliated Hospital of Southwest Medical University have accepted the decision of the ethical committee of West China Hospital, Sichuan University. The written informed consent was waived by the ethical committee for this retrospective study

### Data and image acquisition

Histological sections and CP data were obtained from the corresponding hospitals and delivered to the central laboratory for a unified process. Sections of HE-stained, formalin-fixed, paraffin-embedded breast cancer biopsies were manually reviewed to exclude cases with tissue-processing artifacts or poor staining. Eligible sections were digitally scanned at 40 × magnification using a Hamamatsu scanner (NanoZoomer-XR C12002, Hamamatsu, Japan). Clinical variables, such as age at diagnosis, tumor size, and clinical lymph node status, were gathered from the medical records at each institution, and pathological indicators, including ER, PR, HER2, and Ki67 results, were collected from the pathological diagnostic reports. No less than 1% of positive cells for ER/PR IHC examination was defined as ER/PR positive, and ER- and/or PR-positive breast cancer was classified into HR+ disease. For HER2 status, IHC 3+ and/or amplification by fluorescence in situ hybridization (FISH) were regarded as positive; otherwise, IHC 0/1+ and IHC 2+ with no amplification by FISH indicated HER2-negative (HER2−) disease. According to ref. ^34^, 20% was set as the cutoff point for Ki67, which grouped the patients into a high expression cohort and a low expression cohort. The pathological response to NAC was reviewed at the center laboratory, and the patients were classified into a pCR group and a non-pCR group at each hospital. Here, pCR referred to the elimination of invasive tumor cells at the primary breast site (ypT0).

### Pathological evaluation

Stromal TILs (sTILs) was assessed following the international recommendation guidelines^35^. In brief, all stromal mononuclear cells within the tumor border, including lymphocytes and plasma cells but not macrophages and neutrophils, were counted, and the percentage of sTILs was estimated as a semi-quantitative continuous parameter indicating the density of sTILs. In addition, sTILs was categorized into three grades: low (≤10%), moderate (11–39%), and high (≥40%)^35^. Nuclear grade was assessed based on the Nottingham grading system. Additionally, stromal type classification was performed at the patch level and WSI level by two well-trained observers following the criteria described in previous studies^31^. According to the main component of the stroma, patches/WSIs were classified into the C type, F type, and L type; cases that did not fall into one of the three categories were categorized as the unclassified type. The sTILs, nuclear grade, and stromal type were evaluated on the digital images by two independent observers at the center laboratory, and inconsistent cases were reviewed to reach a consensus.

### ROI annotation

We developed a customized image processing pipeline consisting of three main steps: annotation of ROI, training, and employment of E-S classifier, and TS-score development (Fig. 2).

In this study, we aimed to investigate the predictive potentials of tumor stroma in breast cancer. Unlike the tumor epithelium which contains only tumor cells, stroma is a complex tumor microenvironment that includes not only cells like lymphocytes, fibroblasts, endothelia cells but also non-cellular components forming the extracellular matrix. As tumor stroma is of high heterogeneity across a WSI, ROI selection was of vital importance for investigating the stroma compartment.

Therefore, representative literature related to tumor stromal assessments were reviewed. In some investigations of the tumor stroma, they proposed their standard of the investigated regions. For instance, the tumor-stroma ratio was assessed in some studies^36–40^; in their illustrations, fields were scored where both stroma and tumor were present and tumor cells were seen on all sides of the microscopic image field (north east south west). Similarly, in the study of the correlation between the stromal organization and pathological response to NAC, Dekker et al. thought that only stromal tissues surrounded by tumor cells in each corners were included to the analysis^26^. Moreover, the study of the prognostic value of the stroma morphology in prostate cancer, the stroma regions identified in the representative tumor regions selected by pathologists were used for the experiments^19^, similar to the study of Beck et al^18^. Based on the above reference and the recommended guidelines of tumor-infiltrating lymphocyte^35^, we assumed that only the stroma surrounded by the tumor cells within the representative tumor regions contained predictive information. Representative tumor regions containing tumor stroma were manually annotated on each WSI, ensuring that the stroma inside the ROIs was near the tumor and surrounded by tumor cells19,26,36. Images from ROIs were preprocessed and cropped into 233 × 233 μm squares (256 × 256 pixels at 10 × magnification) called “tiles”.

### Training and employment of the E-S classifier

For the training data, two annotation strategies for tumor epithelium were used by a well-trained pathological observer to better train the model while using less manual efforts^41^, which were as follows: (1) 105 WSIs were roughly annotated to produce noisy sample one (*NS_1*), detail descriptions as follows: annotate the tumor cells inside the black rectangle regions in yellow, ensuing that all tumor cells were included in the annotations and allowing some false annotations (mainly referring the stroma was inside the epithelium annotations) (see Supplementary Fig. 1); (2) 20 WSIs were precisely but partially annotated to produce noisy sample two (*NS_2*), detail descriptions as follows: annotate the tumor cells inside the black rectangle regions in yellow, ensuring all the annotated areas were exactly the tumor epithelium and no need to annotate all the tumor cells (indicating that some tumor cells could be missed for annotations) (see Supplementary Fig. 1).

*NS_1* contains 992 pairs of images and corresponding noisy labels and *NS_2* contains 142 pairs of images and corresponding noisy labels. We also prepared a knowledge base (KB) which contains a list of semantic descriptions for tumor segmentation task in pre-treatment HE-stained biopsied images (Supplementary Fig. 2). The prepared *NS_1*, *NS_2* and KB were employed to train an image semantic segmentation model for the task of identifying the tumor stroma. Images were cropped into 256 × 256 pixels (width × height) at 10 × magnification (called “tile”/patch)^41^.

We employed one-step abductive multi-target learning with diverse noisy samples (OSAMTL-DNS) to learn the labeled noise samples more effectively^41^, as shown in Supplementary Fig. 2. OSAMTL-DNS inherited the original one-step abductive multi-target learning (OSAMTL)^42^ and extended it to handle different noise samples. OSAMTL-DNS consists of three main sections: one-step abductive logical reasoning with diverse noisy samples (OSALR-DNS), target rearrangement, and multi-target learning. More details of the implementations of OSAMTL-DNS are provided in ref. ^41^.

### OSALR-DNS

With the given NS_1, NS_2, and KB, OSALR-DNS, which consists of four sub-steps, abduces multiple targets containing information were consistent with the domain knowledge about the true target of the tumor segmentation task in pre-treatment H&E-stained biopsy images (as shown the green section in the Supplementary Fig. 2)

### Target rearrangement

The target rearrangement step takes the multiple targets produced by OSALR-DNS as input and produce ordered multiple targets that are corresponding to each of the two given NS_1 and NS_2 (as shown in the blue section in Supplementary Fig. 2).

### Multi-target learning

On the basis of the rearranged targets *t* ~ (the binary image of the blue frame in Supplementary Fig. 2) and the target prediction *t* (the binary image of the red frame in Supplementary Fig. 2) of the DCNN architecture, we employ cross entropy (CE) to implement the multi-target learning procedure by Eq. (1)

1 $${{{\mathcal{L}}}}\left( {t,\widetilde t;{\rm{CE}}} \right) = \frac{1}{n}\mathop {\sum}\nolimits_{j = 1}^n {\left( {\alpha _1{\rm{CE}}\left( {t_j,\widetilde t_{j,1}} \right) + \alpha _2{\rm{CE}}\left( {t_j,\widetilde t_{j,2}} \right)} \right)\,{\rm{s.t.}}\,\alpha _1 + \alpha _2 = 1}$$

To optimized the parameters of the DCNN architecture, we employ stochastic gradient descent (SGD) to implement the objective by Eq. (2)

2 $$\mathop {{\min }}\nolimits_i \left( {{{{\mathcal{L}}}}\left( {t,\widetilde t;{\rm{CE}}} \right);{\rm{SGD}}} \right)$$

(as shown in the red section in Supplementary Fig. 2)

With the three procedures done, a E-S classifier for identifying tumor stroma (regions out of belonging to epithelium were deemed as the tumor stroma in the ROIs) was developed.

Based on the training process, an E-S classifier was generated, which could be employed to identify the tumor stroma regions inside the ROIs (as shown the inference part in Fig. 2S). The test set of 19 WSIs were accurately annotated by the pathology expert to produce a relatively noisy-free dataset (RNFS) (also called ground-truth). RNFS contains 158 pairs of images and corresponding accurate labels, among which 79 pairs are for validation and 79 pairs are for testing. We employed the validation set to select the best segmentation model and used the testing set to evaluate the generalization of the selected model. The performance of E-S classifier (CNN I) in the validation and testing set was shown in the Supplementary Table 2. Furthermore, Supplementary Fig. 3 shows the confusion matrix of the E-S classifier for identifying the stroma.

### The development of TS-score

Among the total of 1035 WSIs, 55,078 tiles were cropped from the ROIs. The E-S classifier was used to identify the stroma inside the ROIs. To check the stroma tiles prepared for the following experiments were in the correct classification, a well-trained human observer carefully reviewed all tiles with regions predicted as epithelium or stroma by CNN I (E-S classifier). Meanwhile, IHC sections of CK5/6 and P63 were used as the reference standard if necessary. Tiles containing misclassified stroma, blank regions, and necrosis (see Supplementary Fig. 4) were removed. Finally, 10,344 tiles were excluded and the rest 44,734 were remained.

To develop a stroma-based biomarker for predicting pCR, Inception-V4 was selected as the base DL architecture^43^. Weighted cross-entropy^44^ and stochastic gradient descent (SGD)^45^ were used in optimization. Moreover, we used the fast ensemble DL strategy to further boost the optimization of the prediction part of CNN II^46–48^. After scoring all tiles, an averaged value from all the tiles of each WSI was calculated as the TS-score, which reflected the probability of obtaining pCR for an individual patient (Fig. 2 and Supplementary Fig. 5).

### Statistical analysis

Comparisons among cohorts and between the pCR and non-pCR groups were made with the Pearson χ^2^ test or Fisher’s test for qualitative variables (Table 1 and Supplementary Table 4), while the t test or Mann–Whitney U test was used for continuous variables (Fig. 5). Univariate and multivariate logistic regression methods were used to investigate the correlations between factors and pCR in the PC and VCs. AUCs and 95% confidence interval (95% CI) were used to evaluate the prediction performance, and the DeLong test was used to compare the difference between AUCs^49^. The AUC of bootstrap analysis (100 repetitions) was calculated to estimate the CI in the validations, while 5-fold cross validation was used in the PC. All statistical analyses were two-sided, and a *P* value less than 0.05 indicated statistical significance. The statistical analyses were performed using SPSS software, version 25.

### Reporting summary

Further information on research design is available in the Nature Research Reporting Summary linked to this article.

## Supplementary information

Supplementary Materials

Reporting Summary

## Data Availability

Data used and/or analyzed during the current study are available from the corresponding author on reasonable request.

## Code availability

Python implementation of the TS-score pipeline is maintained in GitHub repository: https://github.com/YongQuanYang/TS-Score.
