# Supplementary material for: Predicting neoadjuvant chemotherapy benefit using deep learning from stromal histology in breast cancer
Source: NPJ Breast Cancer. 2022 Nov 22;8:124. doi: 10.1038/s41523-022-00491-1 (PMC9684483; doi:10.1038/s41523-022-00491-1)
Supplement: Supplementary file 1 — Supplementary Materials [file 41523_2022_491_MOESM1_ESM.pdf]

Supplementary Figures and Tables

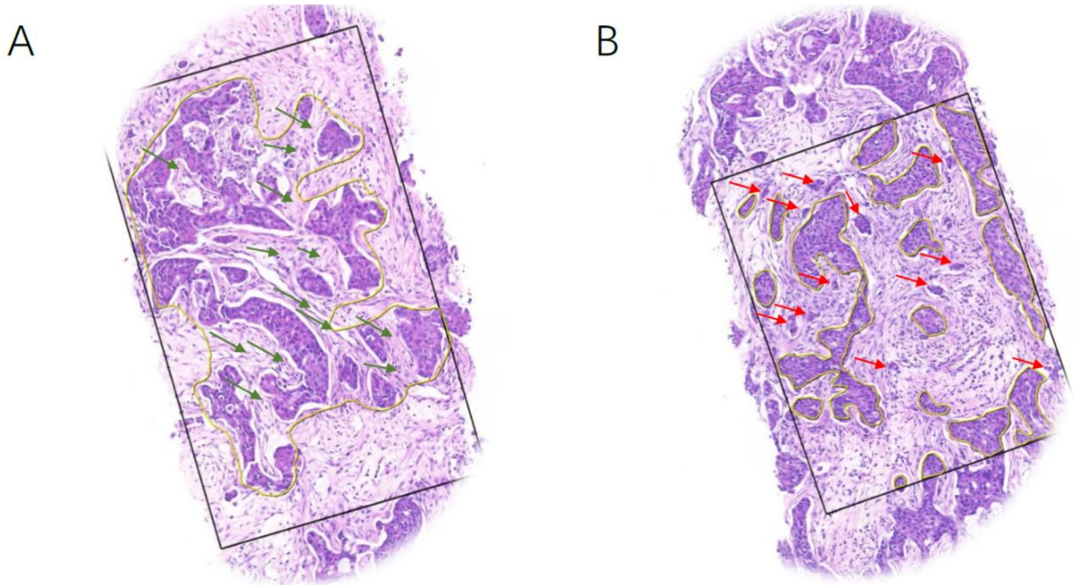

Supplementary Figure 1: Two annotation strategies for tumor epithelium were used to better train the E-S classifier. The black rectangle areas were the representative tumor tissues, and yellow lines circled the regions of tumor epithelium while regions outside the yellow lines were deemed as the stroma. A. An example for rough annotation, green arrows indicated the false annotations, mainly referring the stroma was inside the epithelium annotations. B. An example for precise but partial annotation, red arrows indicated the tumor cells of missed annotations.

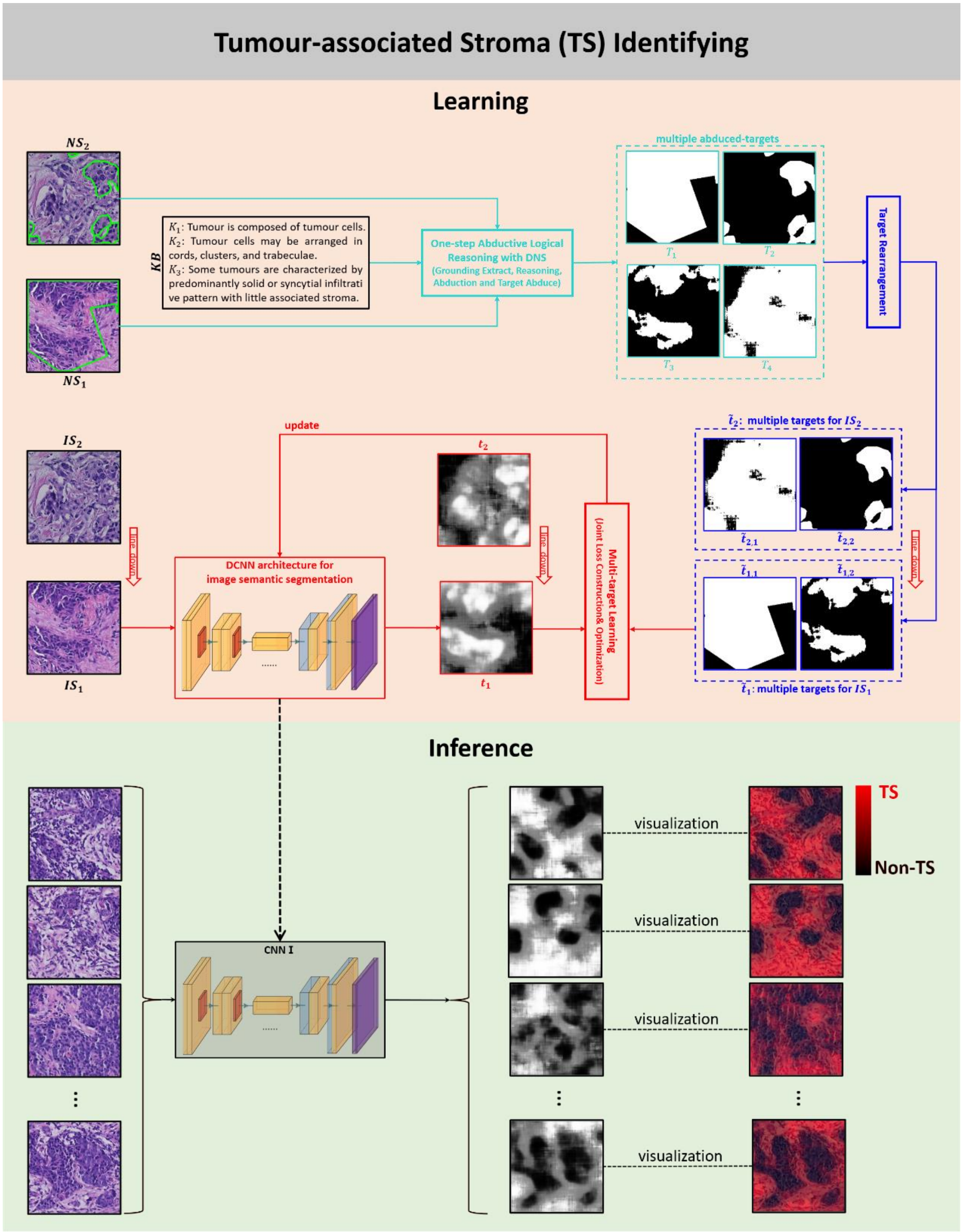

Supplementary Figure 2. The work pipeline of the E-S classifier.

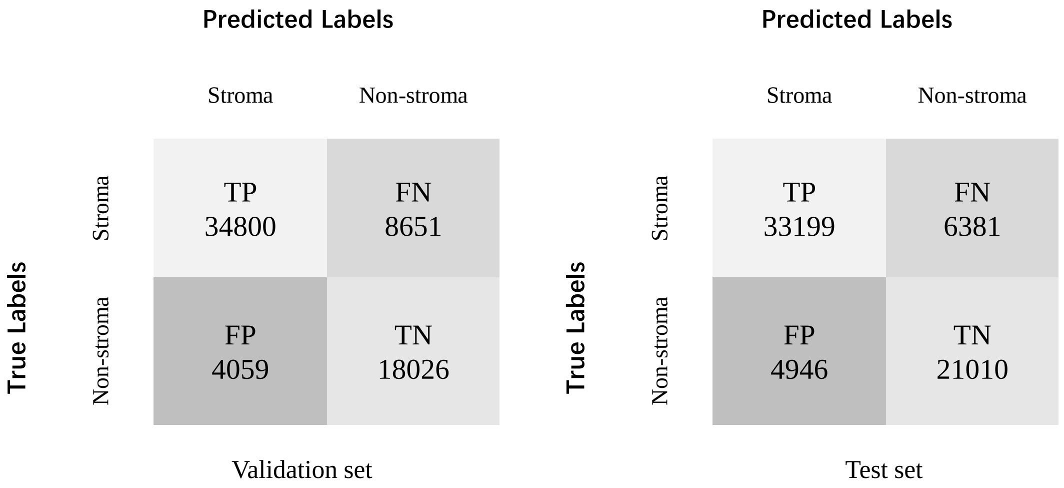

Supplementary Figure 3 Confusion matrix of the E-S classifier for identifying the stroma. The values were referred the amount of pixels.

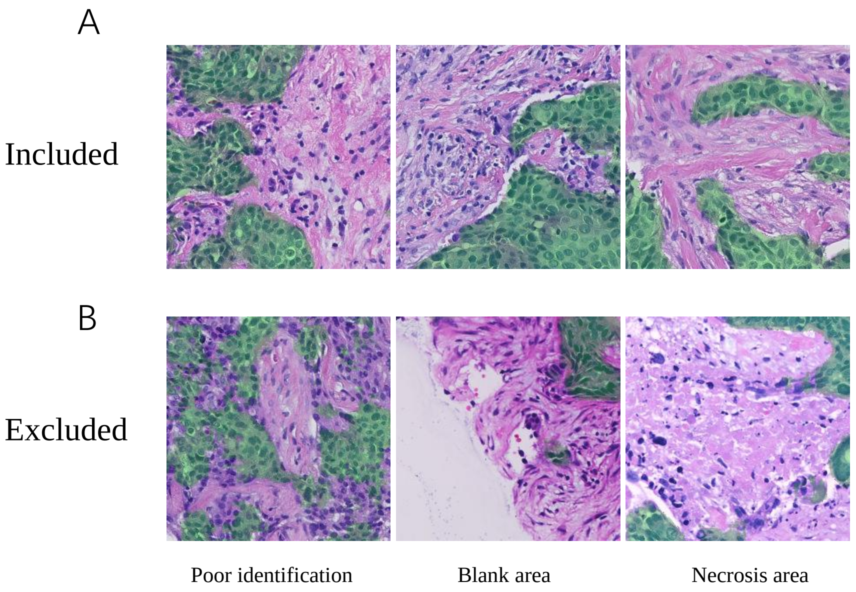

Supplementary Figure 4: Examples of the identified tiles by the E-S classifier, the green regions represent the areas which were identified as tumor while the regions without color mask were identified as stroma. A shows the patches with accurate identification of stroma and these patches were enrolled in our study while B shows the patched with incorrect identification of stroma, lots of blank areas, and necrosis areas and these were remove in this study

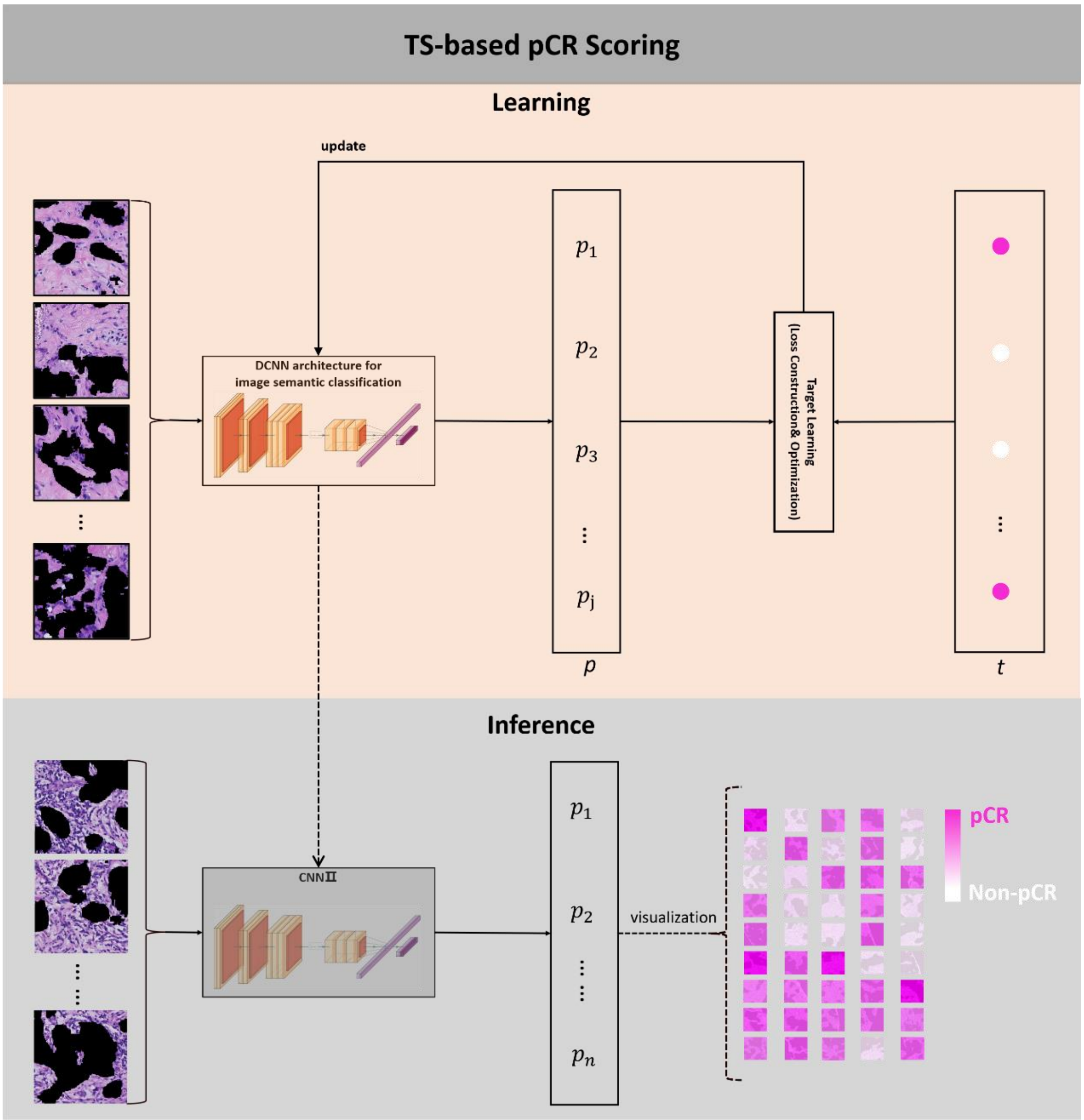

Supplementary Figure 5: The work pipeline of the E-S classifier for generating a TS-score.

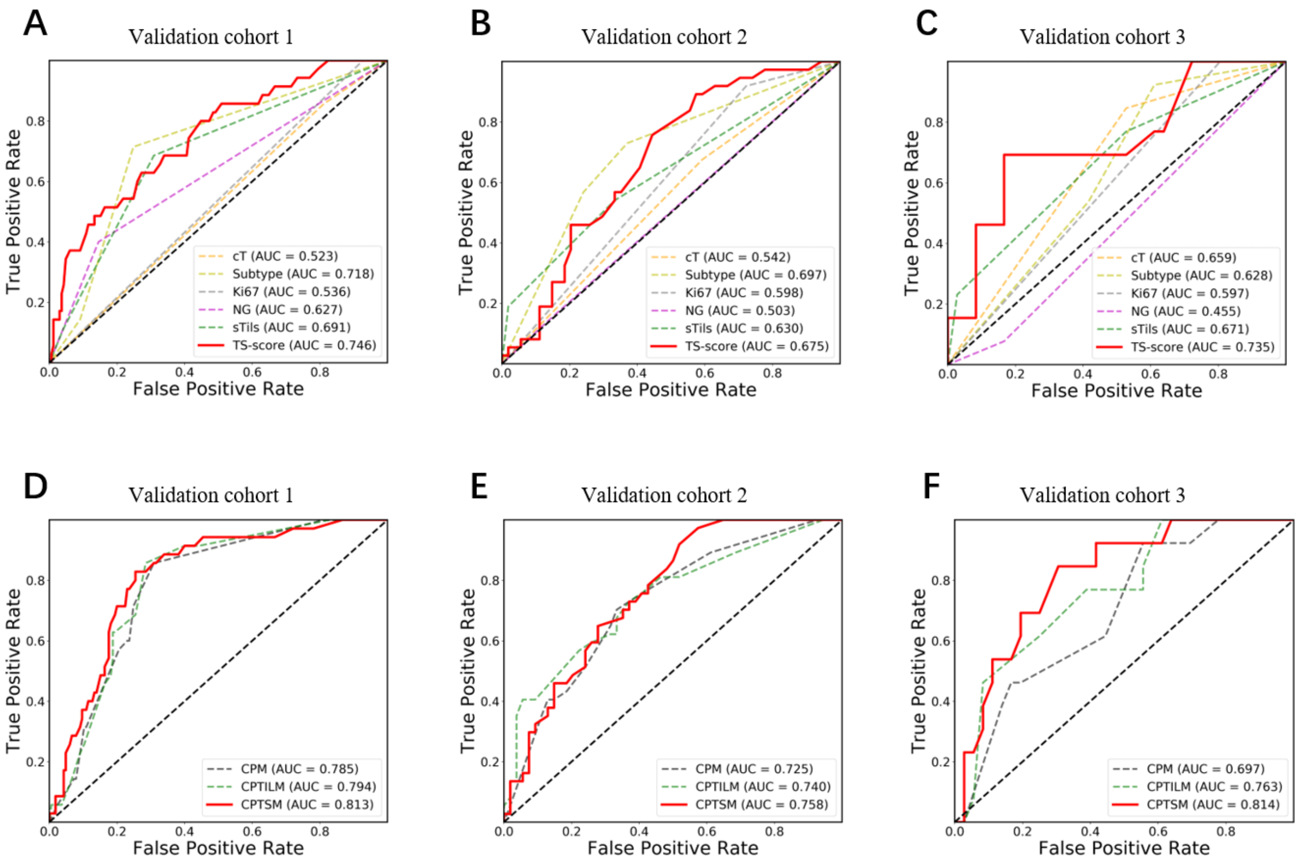

Supplementary Figure 6 ROC curves of the marker-based models. The top row shows the performance of the single marker-based models for predicting pCR in three validations (A, B, C). The bottom row shows the performance of the baseline marker-based model (CPM), the baseline marker and sTILs-based model (CPTILM), and the baseline marker and TS-score-based model (CPTSM) for predicting pCR in three three external validations (D, E, F).

Supplementary Table 1 Inclusion and exclusion notes for patient recruitment

	Inclusion	Exclusion
1	patients with primary invasive ductal breast cancer	patients received a nonstandard treatment regimen, mainly referring to the treatment of HER2+ breast cancers without trastuzumab
2	patients without distant metastasis	patients lacking complete clinical and pathological information
3	patients receiving four, six, or eight cycles of anthracycline and/or taxane-based NAC regimens, and patients with human epidermal growth factor receptor 2-positive (HER2+) diseases who underwent targeted HER2 therapy	patients with HE-stained slides of poor quality, including tissue-processing artifacts such as bubbles, discoloration and soiling caused by long storage time and low tissue volume
4	patients who had undergone surgical treatment after NAC	patients diagnosed with bilateral, multifocal, or special invasive breast cancer

Supplementary Table 2 NAC regimens

NAC classification	NAC regimens	WC (N=695)	SX (N=200)	SC (N=91)	SW (N=49)	Total (N=1035)
Sequential regimens	AC→P/T (+H/HP)	17	16	-	3	175(16.9%)
	EC→T/P (+H/HP)	124	10	2	3	
Combined regimens	TA/C (+H/HP)	9	88	8	7	566(54.7%)
	TE/C (+H/HP)	209	33	29	23	
	PA/C (+H/HP)	21	7	11	-	
	PEC(+H/HP)	113	5	3	-	
Other regimes	TC,EC, AC,FEC (+H/HP)	202	41	38	13	294(28.4%)

Abbreviations: NAC, neoadjuvant chemotherapy; WC, West China hospital; SX, Shanxi Cancer hospital; A, doxorubicin; C, cyclophosphamide; P, paclitaxel; T, docetaxel; E, Epirubicin; H, Herceptin; HP, herceptin and pertuzumab.

Supplementary Table 3 The performance of E-S classifier for identification of stroma.

Dataset	Precision	Recall	F1	fIOU	Acc
Validation	89.55	80.09	84.46	73.25	80.60
Test	87.03	83.88	85.43	74.56	82.71

Abbreviations: fIOU, foreground intersection over union; Acc, accuracy

Supplementary Table 4 Demographic comparisons between pCR and non-pCR groups among primary and validation cohort.

Factors	PC			V1			V2			V3		
	pCR (n=169)	Non-pCR (n=526)	*P*	pCR (n=35)	Non-pCR (n=165)	*P*	pCR (n=37)	Non-pCR (n=54)	*P*	pCR (n=13)	Non-pCR (n=36)	*P*
Age (%)			0.245			0.698			0.277			0.332
<50	91(53.8)	311(59.1)		11(31.4)	60(36.4)		18(48.6)	19(35.2)		5(38.5)	21(58.3)	
≥50	78(46.2)	215(40.9)		24(68.6)	105(63.6)		19(51.4)	35(64.8)		8(61.5)	15(41.7)	
cT (%)			**0.006**			0.634			0.51			0.053
T1-T2	97(57.4)	237(45.1)		30(85.7)	134(81.2)		25(67.6)	32(59.3)		11(84.6)	19(52.8)	
T3-T4	72(42.6)	289(54.9)		5(14.3)	31(18.8)		12(32.4)	22(40.7)		2(15.4)	17(47.2)	
cN (%)			0.754			0.462			0.243			0.733
N0	13(7.7)	45(8.6)		8(22.9)	27(16.4)		8(21.6)	19(35.2)		5(38.5)	11(30.6)	
N1-N3	156(92.3)	481(91.4)		27(77.1)	138(83.6)		29(78.4)	35(64.8)		8(61.5)	25(69.4)	
HR (%)			**<0.001**			**<0.001**			**0.001**			0.172
Negative	89(52.7)	120(22.8)		22(62.9)	37(22.4)		17(45.9)	7(13.0)		7(53.8)	10(27.8)	
Positive	80(47.3)	406(77.2)		13(37.1)	128(77.6)		20(54.1)	47(87.0)		6(46.2)	26(72.2)	
HER2 (%)			**<0.001**			0.542			**0.002**			0.525
Negative	68(40.2)	411(78.1)		30(85.7)	149(90.3)		16(43.2)	41(75.9)		6(46.2)	21(58.3)	
Positive	101(59.8)	115(21.9)		5(14.3)	16(9.7)		21(56.8)	13(24.1)		7(53.8)	15(41.7)	
Subtype (%)			**<0.001**			**<0.001**			**0.002**			0.098
HR+/HER2-	32(18.9)	338(64.3)		10(28.6)	124(75.2)		10(27.0)	34(63.0)		1(7.7)	14(38.9)	
HER2+	101(59.8)	115(21.9)		5(14.3)	15(9.1)		21(56.8)	13(24.1)		7(53.8)	15(41.7)	
TNBC	36(21.3)	73(13.9)		20(57.1)	26(15.8)		6(16.2)	7(13.0)		5(38.5)	7(19.4)	
Ki67 (%)			**0.002**			0.131			0.067			0.167
≥20%	157(92.9)	438(83.3)		35(100.0)	153(92.7)		33(89.2)	39(72.2)		13(100.0)	29(80.6)	
<20%	12(7.1)	88(16.7)		0	12(7.3)		4(10.8)	15(27.8)		0	7(19.4)	
NG(%)			**<0.001**			**0.001**			0.318			0.658
1月2日	91(53.8)	395(75.1)		21(60.0)	141(85.5)		32(86.5)	47(87.0)		12(92.3)	30(83.3)	
3	78(46.2)	131(24.9)		14(40.0)	24(14.5)		5(13.5)	7(13.0)		1(7.7)	6(16.7)	
sTILs (%)			**<0.001**			**<0.001**			**0.011**			**0.045**
Low	60(35.5)	337(64.1)		11(31.4)	114(69.1)		17(45.9)	36(66.7)		3(23.1)	17(47.2)	
Moderate	82(48.5)	169(32.1)		20(57.2)	44(26.7)		13(35.1)	17(31.5)		7(53.8)	18(50.0)	
High	27(16.0)	20(3.8)		4(11.4)	7(4.2)		7(18.9)	1(1.9)		3(23.1)	1(2.8)	

Abbreviations: HR, hormone receptor; HER2, human epidermal growth factor receptor 2; sTILs, stromal tumor-infiltrating lymphocytes; NG, nuclear grade; pCR, pathological complete response.

Bold indicates statistical significance ($P < 0.05$)

Supplementary Table 5. AUCs of TS-score with separation by breast cancer subtypes at WSI-level.

	PC (95% CI)	V1 (95% CI)	V2 (95% CI)	V3 (95% CI)
HR+HER2-	0.767 (0.688-0.846)	0.804 (0.784-0.824)	0.784 (0.771-0.797)	1.00 (0.910-1.00)
HER2+	0.675 (0.595-0.755)	0.725 (0.701-0.749)	0.581 (0.559-0.603)	0.652 (0.628-0.676)
TNBC	0.701 (0.647-0.755)	0.541 (0.525-0.557)	0.536 (0.501-0.571)	0.743 (0.705-0.781)
Total	0.729 (0.714-0.744)	0.745 (0.737-0.753)	0.673 (0.662-0.684)	0.725 (0.708-0.742)

Supplementary Table 6. AUCs of TS-score with separation by breast cancer subtypes at patch-level.

	PC (95% CI)	V1 (95% CI)	V2 (95% CI)	V3 (95% CI)
HR+HER2-	0.684 (0.610-0.758)	0.765 (0.753-0.777)	0.659 (0.651-0.667)	0.898 (0.818-0.978)
HER2+	0.610 (0.578-0.642)	0.700 (0.0.679-0.721)	0.477 (0.464-0.490)	0.648 (0.631-0.665)
TNBC	0.621 (0.577-0.665)	0.530 (0.521-0.539)	0.558 (0.544-0.572)	0.693 (0.670-0.716)
Total	0.654 (0.626-0.682)	0.695 (0.689-0.701)	0.599 (0.590-0.607)	0.676 (0.662-0.689)

Supplementary Table 7 Univariate and multivariate analysis of TS-score correlating with pCR in the validation cohorts.

Factors	Univariate analysis		Multivariate analysis [1]		Multivariate analysis [2]	
	OR(95% CI)	*P*	OR(95% CI)	*P*	OR(95% CI)	*P*
TS-score	-	**<0.001**	-	**-**	-	**0.013**
Subtypes	-	**<0.001**	-	**<0.001**	-	**<0.001**
HR+/HER2-	1	-	1	-	1	-
HER2+	6.29(3.31-11.9)	**<0.001**	5.71(2.81-11.6)	**<0.001**	7.02(3.35-14.7)	**<0.001**
TNBC	6.35(3.30-12.2)	**<0.001**	5.49(2.70-11.2)	**<0.001**	5.40(2.64-11.0)	**<0.001**
sTILs	-	**<0.001**	-	**0.012**	-	0.584
Low	1	-	1	-	1	-
Moderate	2.73(1.59-4.68)	**<0.001**	1.92(1.05-3.53)	**0.035**	1.21(0.59-2.47)	0.599
High	8.38(3.34-21.0)	**0.002**	4.34(1.51-12.5)	**0.007**	1.92(0.56-6.56)	0.300
cT	1.31(0.74-2.35)	0.355	0.65(0.33-1.27)	0.206	0.72(0.37-1.42)	0.343
Ki67	4.02(1.20-13.5)	**0.024**	2.35(0.63-8.77)	0.204	2.15(0.56-8.32)	0.268
NG	1.81(0.98-3.34)	0.056	1.03(0.49-2.16)	0.930	0.96(0.45-2.04)	0.915

Note: Multivariate analysis [1] refers to the multivariate analysis excluding BCSS; Multivariate analysis [2] refers to the multivariate analysis including the BCSS.

Abbreviation: HR, hormone receptor; HER2, human epidermal growth factor receptor 2; sTILs, stromal tumor-infiltrating lymphocytes; NG, nuclear grade

Bold indicates statistical significance ($P < 0.05$)

Supplementary Table 8. AUCs of single marker-based model for pCR prediction in primary and validation cohorts

Model	PC (95% CI)	*P*	VCs (95% CI)	*P*	V1 (95% CI)	*P*	V2 (95% CI)	*P*	V3 (95% CI)	*P*
TS-score	0.727 (0.711-0.743)	1	0.681 (0.675-0.687)	1	0.746 (0.736-0.756)	1	0.675 (0.664-0.686)	1	0.735 (0.717-0.753)	1
sTILs	0.651 (0.611-0.691)	**<0.001**	0.622 (0.616-0.628)	0.517	0.691 (0.682-0.700)	**0.036**	0.630 (0.619-0.641)	0.420	0.671 (0.654-0.688)	0.419
Subtype	0.727 (0.689-0.765)	0.927	0.713 (0.708-0.718)	0.490	0.718 (0.709-0.727)	0.631	0.697 (0.686-0.708)	0.759	0.628 (0.614-0.642)	0.408
cT	0.546 (0.513-0.579)	**<0.001**	0.525 (0.519-0.531)	**<0.001**	0.523 (0.516-0.530)	**<0.001**	0.542 (0.531-0.551)	0.081	0.659 (0.645-0.673)	0.434
Ki67	0.547 (0.532-0.562)	**<0.001**	0.549 (0.546-0.552)	**<0.001**	0.536 (0.534-0.538)	**<0.001**	0.598 (0.590-0.606)	0.252	0.597 (0.590-0.604)	0.111
NG	0.592 (0.566-0.622)	**<0.001**	0.545 (0.540-0.550)	**<0.001**	0.627 (0.617-0.637)	**0.032**	0.503 (0.496-0.510)	0.007	0.455 (0.445-0.465)	**0.002**

Abbreviations: AUC, area under receiver operating characteristic curve; *pCR*, pathological complete response;

p value refers to Delong test for the differences of AUCs between different metrics

[*] $p < 0.05$, with significant or marginally significant differences for AUCs of … compared with that of TS-score

Supplementary Table 9. AUCs of models for pCR prediction in the primary and validation cohorts

		CPTSM (95% CI)	CPTILM (95% CI)	CPM (95% CI)
V1		0.813 (0.806-0.820)	0.794 (0.788-0.800)	0.785 (0.778-0.792)
	p value[1]	0.265	0.661	1
	p value[2]	0.199	1	-
V2		0.758 (0.749-0.767)	0.740 (0.728-0.751)	0.725 (0.714-0.736)
	p value[1]	0.204	0.237	1
	p value[2]	0.614	1	-
V3		0.814 (0.801-0.827)	0.763 (0.749-0.777)	0.697 (0.682-0.712)
	p value[1]	0.139	0.235	1
	p value[2]	0.313	1	-

Abbreviations: AUC, area under receiver operating characteristic curve; pCR, pathological complete response; CPM, clinicopathology-based model; CPTILM, clinicopathology and sTILs based model; CPTSM, clinicopathology and TS-score based model.

p value refers to Delong test for the differences of AUCs between different metrics in different cohorts; p value1 refers the comparisons with the CPM while p value2 refers the comparisons with CPTILM.

Bold indicates statistical significance ($P < 0.05$)

Supplementary Table 10 The distributions of stromal histological patterns in pCR and non-pCR groups

Group	C	F	L	U	Sum
pCR	47 (18.5%)	72 (28.3%)	112 (44.1%)	23 (9.1%)	254
Non-pCR	284 (36.4%)	278 (35.6%)	154 (19.7%)	65 (8.3%)	781
Total	331 (32.0%)	350 (33.8%)	266 (25.7%)	88 (8.5%)	1035
pCR rate	14.2%	20.6%	42.1%	26.1%	24.5%
