# Supplementary material for: Predicting neoadjuvant chemotherapy benefit using deep learning from stromal histology in breast cancer
Source: NPJ Breast Cancer. 2022 Nov 22;8:124. doi: 10.1038/s41523-022-00491-1 (PMC9684483; doi:10.1038/s41523-022-00491-1)
Supplement: Supplementary file 2 — Reporting Summary [file 41523_2022_491_MOESM2_ESM.pdf]

nature portfolio

Corresponding author(s): Zhongxi Zheng, Hong Bu

Last updated by author(s): Aug 10, 2022

Reporting Summary

Nature Portfolio wishes to improve the reproducibility of the work that we publish. This form provides structure for consistency and transparency in reporting. For further information on Nature Portfolio policies, see our Editorial Policies and the Editorial Policy Checklist.

Statistics

For all statistical analyses, confirm that the following items are present in the figure legend, table legend, main text, or Methods section.

n/a	Confirmed	
☐	☒	The exact sample size (*n*) for each experimental group/condition, given as a discrete number and unit of measurement
☐	☒	A statement on whether measurements were taken from distinct samples or whether the same sample was measured repeatedly
☐	☒	The statistical test(s) used AND whether they are one- or two-sided<br>*Only common tests should be described solely by name; describe more complex techniques in the Methods section.*
☐	☒	A description of all covariates tested
☐	☒	A description of any assumptions or corrections, such as tests of normality and adjustment for multiple comparisons
☐	☒	A full description of the statistical parameters including central tendency (e.g. means) or other basic estimates (e.g. regression coefficient) AND variation (e.g. standard deviation) or associated estimates of uncertainty (e.g. confidence intervals)
☐	☒	For null hypothesis testing, the test statistic (e.g. *F*, *t*, *r*) with confidence intervals, effect sizes, degrees of freedom and *P* value noted<br>*Give P values as exact values whenever suitable.*
☒	☐	For Bayesian analysis, information on the choice of priors and Markov chain Monte Carlo settings
☐	☒	For hierarchical and complex designs, identification of the appropriate level for tests and full reporting of outcomes
☐	☒	Estimates of effect sizes (e.g. Cohen's *d*, Pearson's *r*), indicating how they were calculated

Our web collection on statistics for biologists contains articles on many of the points above.

Software and code

Policy information about availability of computer code

Data collection	no software was used to collect data. Microsoft Office Excel 2016 was used to clean and organize data and NDP.View 2 was used to review and annotate images.
Data analysis	Data analysis was done by python 3.5, tensorflow 2.3.0/1.10.0 and IBM SPSS Statistics 25.

For manuscripts utilizing custom algorithms or software that are central to the research but not yet described in published literature, software must be made available to editors and reviewers. We strongly encourage code deposition in a community repository (e.g. GitHub). See the Nature Portfolio guidelines for submitting code & software for further information.

Data

Policy information about availability of data

All manuscripts must include a data availability statement. This statement should provide the following information, where applicable:

- Accession codes, unique identifiers, or web links for publicly available datasets
- A description of any restrictions on data availability
- For clinical datasets or third party data, please ensure that the statement adheres to our policy

The data for this study is available from the corresponding author on reasonable request and the code can be found at the GitHub for this paper:https://github.com/YongQuanYang/TS-Score

Human research participants

Policy information about studies involving human research participants and Sex and Gender in Research.

Reporting on sex and gender	In this study, we recruited 1035 breast cancer patients from four hospitals and all of them are females. Breast cancer cases in men are relatively rare compared to women;hence, our study concentrated on female breast cancers.
Population characteristics	In this study, we recruited 1035 breast cancer patients from four Chinese hospitals and they were diagnosed with invasive ductal carcinoma and without distant metastasis. All patients received neoadjuvant chemotherapy before surgery.
Recruitment	All patients were recruited under strict screening criteria.The inclusion criteria were as follows: 1) patients with primary invasive ductal breast cancer; 2) patients without distant metastasis; 3) patients receiving four, six, or eight cycles of anthracycline and/or taxane-based NAC regimens, and patients with human epidermal growth factor receptor 2-positive (HER2+) diseases who underwent targeted HER2 therapy (NAC regimens are detailed in Table S1); and 4) patients who had undergone surgical treatment after NAC. On the other hand, patients with HE-stained slides of poor quality, including tissue-processing artifacts such as bubbles, discoloration and soiling caused by long storage time and low tissue volume, were excluded from our study. In total, 1035 eligible patients were enrolled, and a detailed recruitment flowchart is shown in manuscript. All patients were Chinese.
Ethics oversight	Our study approved by the ethical committee of West China Hospital, Sichuan University (No.764 in 2021), and abided with the Declaration of Helsinki before using tissue samples for scientifc researches purpose only.The other three hospitals, including Shanxi Cancer Hospital, Sichuan Province People's Hospital, and the Affiliated Hospital of Southwest Medical University have accepted the decision of the ethical committee of West China Hospital, Sichuan University.

Note that full information on the approval of the study protocol must also be provided in the manuscript.

Field-specific reporting

Please select the one below that is the best fit for your research. If you are not sure, read the appropriate sections before making your selection.

☒ Life sciences ☐ Behavioural & social sciences ☐ Ecological, evolutionary & environmental sciences

For a reference copy of the document with all sections, see nature.com/documents/nr-reporting-summary-flat.pdf

Life sciences study design

All studies must disclose on these points even when the disclosure is negative.

Sample size	Our study enrolled a total of 1035 patients from four independent institutions: West China Hospital (WC cohort, 695 patients from 2010.04~2021.04), Shanxi Cancer Hospital (SX cohort, 200 patients from 2015.02~2019.10), Sichuan Province People's Hospital (SC cohort, 91 patients from 2020.01~2021.02), and the Affiliated Hospital of Southwest Medical University (SW cohort, 49 patients from 2016.08~2020.10). No sample-size calculation was performed. Due to the time point of sample collection and the preservation of sample quality, the patient recruiting timelines for the four units differed. For our purposes, deep neural network modeling would ideally include as many patients as is practical, as opposed to deciding on an appropriate size. As a result, we did not calculate the sample size.
Data exclusions	According to the strict screening criteria designed for our study, some patients were excluded. The exclusion criteria were as follow: 1)patients received a nonstandard treatment regimen, mainly referring to the treatment of HER2+ breast cancers without trastuzumab; 2)patients lacking complete clinical and pathological information; 3)patients with HE-stained slides of poor quality, including tissue-processing artifacts such as bubbles, discoloration and soiling caused by long storage time and low tissue volume; 4)patients diagnosed with bilateral, multifocal, or special invasive breast cancer. The exclusion criteria were showed in the manuscript.
Replication	The trained neural network is not fully replicable due to small changes that occur during training and the deviations in training data. Re-runs of the network training show that the network performs at the same level between different runs and the difference does not affect the final conclusion.
Randomization	We chose the hospital with the greatest sample size to serve as the training set and the other three hospitals as the validation set since training neural networks requires a substantial quantity of data to ensure the robustness. Slides for five-fold cross validation in training the neural network was performed, as to not bias the network.
Blinding	Investigators were blinded during data collection. Investigators were unblinded to group allocation after analyses were finalized.

Reporting for specific materials, systems and methods

We require information from authors about some types of materials, experimental systems and methods used in many studies. Here, indicate whether each material, system or method listed is relevant to your study. If you are not sure if a list item applies to your research, read the appropriate section before selecting a response.

Materials & experimental systems

n/a	Involved in the study
☒	☐ Antibodies
☒	☐ Eukaryotic cell lines
☒	☐ Palaeontology and archaeology
☒	☐ Animals and other organisms
☒	☐ Clinical data
☒	☐ Dual use research of concern

Methods

n/a	Involved in the study
☒	☐ ChIP-seq
☒	☐ Flow cytometry
☒	☐ MRI-based neuroimaging
